# “Normal vulva” based on the first national Labiagram design in adult Iranian women not seeking female genital cosmetic surgery: a pilot study

**DOI:** 10.1093/sexmed/qfad070

**Published:** 2024-01-19

**Authors:** Zinat Ghanbari, Maryam Kazemi, Nasim Eshraghi, Sina Shiri Hamedani, Azam Zafarbakhsh

**Affiliations:** Department of Obstetrics and Gynecology, Vali-Asr Reproductive Health Research Center, Tehran University of Medical Sciences, Tehran, 1417613151, Iran; Department of Obstetrics and Gynecology, Vali-Asr Reproductive Health Research Center, Tehran University of Medical Sciences, Tehran, 1417613151, Iran; Vali-Asr Reproductive Health Research Center, Family Health Research Institute, Tehran University of Medical Sciences, Tehran, 1419733141, Iran; Department of Pediatrics, School of Medicine, Qazvin University of Medical Sciences, Qazvin, 59811-34197, Iran; Department of Obstetrics and Gynecology, Isfahan University of Medical Sciences, Isfahan, 73461-81746, Iran

**Keywords:** labia minora, Labiagram, cosmetic surgery, female genitalia, vulva

## Abstract

**Background:**

Several studies have been published to present normal values of female genitalia in different age and racial groups.

**Aim:**

The primary objective of our study was to measure the parameters of the external genitalia in adult Iranian women, record the data using the Labiagram system (the first national Labiagram design in Iran), and establish a preliminary database.

**Methods:**

A descriptive study was conducted from March 2022 to December 2022, involving 220 nonpregnant adult women who presented to the gynecology clinic. Women who met the inclusion criteria for the study underwent a comprehensive examination of the external genitalia. The data collected during the examinations were recorded in electronic files and the Labiagram system.

**Outcomes:**

The data showed the diversity of external genital parameters of nonpregnant adult Iranian women.

**Results:**

In this descriptive study, the mean ± SD age of the participants was 51.5 ± 13.44 years, ranging from 15 to 84 years. A total of 192 women (87.3%) had a history of vaginal delivery. There was no statistically significant difference observed in the average measurements of the vulva among the 4 age groups (*P* < .05). The Pearson correlation coefficient test indicated a statistically weak correlation between body mass index and perineum length (*r* = 0.174, *P* = .010). Additionally, a weak correlation was found between body mass index and the width of the labia minora at the left-lower point (*r* = 0.143, *P* = .030) and the right-middle point (*r* = 0.146, *P* = .031). Furthermore, the results demonstrated that women with a history of vaginal delivery had a significantly longer introitus (49.3 vs 44.3 mm, *P* = .037), longer labia majora (91.3 vs 87.3 mm, *P* = .046), and longer labia minora (56.8 vs 50.9 mm, *P* = .008) when compared with women without prior labor experience.

**Clinical Implications:**

The data will be used as a basis for future studies.

**Strengths and Limitations:**

The use of simple tools for the measuring, data recording, and digital drawing of female external genital anatomy, along with privacy protection, is one of the strengths of this research. The weakness is the small sample size, which is the reason for piloting the Labiagram chart for more extensive studies.

**Conclusion:**

Increasing age and the number of births had no statistically significant effect on the size of external genital parameters among Iranian women. Despite the considerable diversity in these parameters, it has not resulted in a significant demand among Iranian women for female cosmetic surgery.

## Introduction

The appearance of a woman’s natural external genitalia exhibits significant variations. Defining what constitutes normal genitalia is subjective, as it is influenced by factors such as ethnicity, age, weight, hormonal status, and skin type.^1^^,^^2^ For instance, while many women express a desire to reduce the size of their labia minora, in Zambian culture, larger labia minora are considered a desirable cosmetic feature of a woman’s genitals.^3^ The literature has documented various forms of female external genitalia (vulva) since 1924, but there is no universally accepted standard for aesthetically perfect labia minora.^4-6^

Researchers are actively working to define and classify hypertrophy and standardize the normal size range of labia minora to reduce the frequency of female genital cosmetic surgery (FGCS) and prevent unnecessary procedures.^7^ The demand for FGCS has risen in recent years as women have become more body conscious. In 2020, during the COVID-19 pandemic, there were 13 697 recorded labiaplasties in the United States.^8^ Despite the increasing number of labiaplasty procedures, there is still limited information available regarding the wide variety of external genitalia morphologies. This lack of information hampers the ability to educate individuals considering such surgery and to evaluate the benefits and risks associated with different surgical treatments.^9^ Among Asian women, cosmetic reasons, followed by pain and discomfort, are reported as the most common motivations for requesting labiaplasty.^10^

FGCS remains ethically controversial in countries where cultural and religious norms restrict the examination of vulvar anatomy. Preserving the modesty and dignity of women is crucial worldwide, not just in Islamic countries. It is important to recognize that attaching a photo before and after labiaplasty in a patient’s file for documentation purposes violates the privacy of the individual. To address this concern, a Labiagram can be employed: a web-based system that generates a schematic diagram of the female external genitalia based on multiple measurements from different genital points. The Labiagram provides a safe and privacy-preserving approach while achieving a high degree of similarity to female genital anatomy.^11^ Given that genital appearance can vary by factors such as ethnicity, age, and weight, the primary objective of our descriptive study was to measure and present the parameters of Iranian women’s external genitalia. How are the features of the vulva in Iranian adult women who do not seek FGCS evaluated according to the first national Labiagram design?

## Methods

We conducted a cross-sectional study at the outpatient clinics of the obstetrics and gynecology department in Imam Khomeini Hospital, Tehran. The study included women who were satisfied with the genital examination and willing to participate. The recruitment period spanned March 2022 to December 2022, and we enrolled Iranian women who were at least 15 years old and willing to provide informed consent. We excluded women who met any of the following criteria: genital ambiguity, current use of systemic hormone therapy, pregnancy, vulvar disease, or prior vulvar surgery. This study received approval from the Ethics Committee of the Tehran University of Medical Sciences (IR.TUMS.IKHC.REC.1401.191).

In this study, we investigated basic characteristics, such as age, body mass index (BMI), number of deliveries, and type of delivery (normal vaginal delivery, cesarean section, or both). The participants were divided into 4 groups based on their age (15-32, 33-49, 50-66, and 67-84 years).

**Figure 1:**
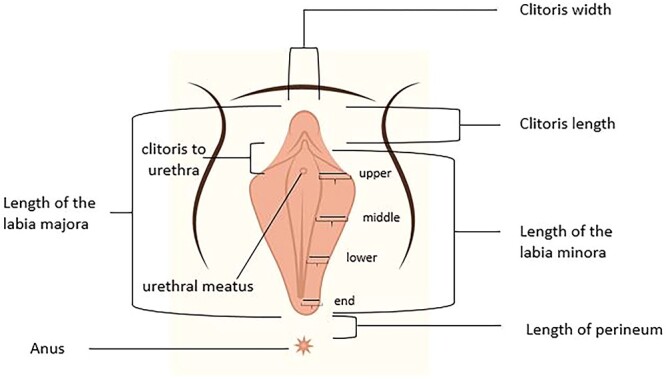
Measured parts on the Labiagram. The width of the labia minora was measured in the upper, middle, lower third, and endpoint (from Hart’s line to the outer edge of the labia minora).

Measurements of the female external genitalia were taken by a female pelvic floor fellow while the participants were in the lithotomy position. A disposable paper measuring tape was used for the measurements. The recorded measurements included the following: length and width of the clitoral glans, distance from the clitoris to the urethral meatus, length of the perineum (from the posterior fourchette to the anterior margin of the anus), length of the labia majora, length of the labia minora (from the clitoris to the lower edge of the labia), width of the left labia minora at 4 points (upper, middle, lower, and endpoint: L1-L4, respectively), and width of the right labia minora at 4 points (upper, middle, lower, and endpoint: R1-R4) from Hart’s line to outer edge with minimal tension). These measurements were recorded in the Labiagram electronic system of the Tehran University of Medical Sciences and stored via Labiagram software^12^ (Figure 1).

The Labiagram system is a web-based platform that can be accessed through various devices, including smartphones, tablets, and computers. It can be deployed on local networks within clinics or as a secure online website on the internet. The system was developed with the C#.Net 2022 programming language, while SQL Server 2019 was utilized for data storage and retrieval from the database.

For statistical analysis, we employed SPSS software (version 26.0; IBM). Mean and SD were used to present the characteristics and measurements of the external genitalia in the participant sample. After normality of the data was confirmed, a 1-way analysis of variance test was conducted to compare the 4 age groups. In cases where the data did not follow a normal distribution, the Kruskal-Wallis test was employed for group comparisons. The significance level was set at *P* < .05.

## Results

This study evaluated a total of 220 women who attended the gynecologic clinic at Imam Khomeini Hospital from March 2022 to December 2022. The demographic characteristics of patients are presented in Table 1. The participants had a mean ± SD age of 51.5 ± 13.44 years, with a range of 15 to 84 years. The average BMI of the participants was 27.30 ± 4.77. The number of women who had experienced vaginal delivery was 192 (87.3%).

**Table 1:** Demographic information of the study participants (N = 220).

**Characteristic**	**Mean ± SD (Range) or No. (%)**
Age, y	51.15 ± 13.44 (15-84)
Body mass index, kg/m^2^	27.30 ± 4.77 (17-42)
Gravidity	
0 or 1	37 (16.81)
≥2	183 (83.18)
Delivery	
Vaginal delivery	161 (73.2)
Cesarean section	18 (8.2)
Cesarean section and normal vaginal delivery	31 (14.1)
Nothing	10 (4.5)
Education	
Illiterate	101 (45.9)
Educated	119 (54.1)

Table 2 presents the vulva measurements in the study participants. The mean length of the introitus was 48.71 ± 11.99 mm; the width of the clitoris was 8.08 ± 2.95 mm and its length was 9.87 ± 3.76 mm; the length of the perineum was 33.50 ± 9.03 mm; and the labia majora length was 90.8 ± 9.98 mm. Table 3 shows the average values of the labia minora width, which did not differ significantly between the left (L1- L4) and right (R1-R4) measurements. The width of the labia minora was highest at the upper level and decreased toward the end.

**Table 2:** External genital measurements of all participants.

	**Measurement, mm**		
	**Mean ± SD (range)**	**Minimum**	**Maximum**
Introitus opening	48.71 ± (11.99)	20	90
Clitoris			
Width	8.08 ± (2.95)	1	18
Length	9.87 ± (3.76)	3	25
Distance: clitoris-urethra	18.80 ± (5.75)	2	40
Length			
Perineum	33.50 ± (9.03)	15	70
Labia majora	90.8 ± (9.98)	60	110
Labia minora	56.02 ± (11.03)	30	80

**Table 3:** Measurements of L1-L4 and R1-R4.

	**Width of labia minora, mm**		
	**Mean**	**Minimum**	**Maximum**
Left			
L1: upper	17.59	3	60
L2: middle	14.58	0	60
L3: lower	4.97	0	30
L4: end	0.52	0	20
Right			
R1: upper	16.72	2	60
R2: middle	13.15	0	50
R3: lower	4.42	0	20
R4: end	0.31	0	10

The study participants were divided into 4 age groups, and the average values of external genitalia measurements for each group are presented in Tables 4 and 5. There were no statistically significant differences in the average vulva measurements among the 4 groups (*P* > .05). These findings suggest that age may not be a significant factor in determining external genitalia measurements in the study population.

**Table 4:** External genital measurements among age groups.

	**Age group, mm**				
	**15-32 y** **(*n* = 15, 6.82%)**	**33-49 y** **(*n* = 87, 39.54%)**	**50-66 y** **(*n* = 86, 39.09%)**	**67-84 y** **(*n* = 32, 14.54%)**	***P* value**
Introitus opening					.968
Mean	40.9	48.3	49.1	44.6	
95% CI	42.5-55.5	45.9-50.8	46.2-52.0	44.6-52.9	
Range	25-70	20-70	20-90	20-70	
Length of labia majora					.975
Mean	90.7	90.9	91.2	90.2	
95% CI	82.7-98.6	88.9-92.9	88.9-93.6	87.1-93.1	
Range	60-110	70-110	60-110	80-110	
Length of labia minora					.250
Mean	50.7	56.7	56.2	57.3	
95% CI	43.6-57.7	54.2-59.2	53.8-58.5	53.6-61.1	
Range	30-70	30-80	30-80	40-80	
Distance: clitoris-urethra					.731
Mean	18.9	19.3	18.5	18.4	
95% CI	14.4-23.4	18.1-20.6	17.3-19.6	16.3-20.5	
Range	10-40	2-30	10-40	5-30	
Length of perineum					.066
Mean	35	33.9	31.7	36.6	
95% CI	28.5-41.4	32.1-35.8	29.9-33.5	32.7-40.5	
Range	20-70	20-60	15-50	15-70	
Length of clitoris					.273
Mean	8.5	9.5	10.3	10.3	
95% CI	6.8-10.1	8.7-10.2	9.4-11.1	8.7-11.8	
Range	3-16	3-20	4-25	5-20	
Width of clitoris					.996
Mean	7.9	8.1	8.2	7.8	
95% CI	6.5-9.3	7.3-8.7	7.6-8.7	6.6-9.1	
Range	4-13	3-18	3-15	1-15	

**Table 5:** Width of the labia minora among age groups.

	**Width of labia minora, mm (mean)**				
	**15-32 y** **(*n* = 15, 6.82%)**	**33-49 y** **(*n* = 87, 39.54%)**	**50-66 y** **(*n* = 86, 39.09%)**	**67-84 y** **(*n* = 32, 14.54%)**	***P* value**
Left					
L1: upper	17.73	17.28	17.98	16.9	.900
L2: middle	15.01	15.96	13.37	15.19	.830
L3: lower	5.6	5.73	4.46	4.87	.579
L4: end	0.67	0.48	0.42	0.97	.230
Right					
R1: upper	15.93	17.33	16.56	15.97	.973
R2: middle	13.4	14.99	11.48	14	.635
R3: lower	4.53	5.17	3.78	4.74	.396
R4: end	0	0.28	0.33	0.55	.580

The statistical analysis of the correlations between BMI and vulva measurements according to the Pearson correlation coefficient test showed a statistically significant but weak correlation between BMI and length of the perineum (*r* = 0.174, *P* = .010), as well as the width of the labia minora at L3 (left-lower point; *r* = 0.143, *P* = .03) and R2 (right-middle point; *r* = 0.146, *P* = .031).

In further analysis, an independent-sample *t*-test or Mann-Whiteney *U* test was used to compare patients who had normal vaginal delivery (*n* = 192, 87.3%) with those who had not (*n* = 28, 12.7%). The results showed that women who had a normal vaginal delivery had significantly longer introitus (49.3 vs 44.3 mm, *P* = .037), longer labia majora (91.3 vs 87.3 mm, *P* = .046), and longer labia minora (56.8 vs 50.9 mm, *P* = .008) when compared with women who had not experienced a normal vaginal delivery.

## Discussion

The appearance of female genital organs can vary by factors such as age, weight, and race.^2^^,^^3^^,^^13-15^ However, measuring and presenting genital parameters in certain countries can be challenging—particularly those with cultural and religious considerations, such as Islamic countries. To address these challenges and promote informed decision making among patients seeking labiaplasty while minimizing unnecessary surgery, the Labiagram schematic diagram has been introduced.

This study showed a weak correlation between BMI and perineal length, which differs from the study conducted by Kreklau et al. They found an inverse correlation between age and length of the labia minora and perineum, as well as a positive correlation between BMI and length of the labia majora and introitus.^13^ Additionally, we found that the length of the labia majora, labia minora, and introitus was greater in women who had a history of vaginal delivery as compared with others. This difference may be attributed to the possibility of pelvic organ prolapse and perineal prolapse in women with a history of vaginal delivery.

The width of the labia minora did not show a significant difference between women >50 years old and younger individuals. This finding contradicts previous studies that indicated wider labia minora in premenopausal women when compared with postmenopausal women. However, it is important to note that these previous studies measured the labia minora at different points, which could contribute to the disparity in results.^16^ Che et al found that accurate comparisons of genital dimensions can be achieved through the digital conversion of photographs. They divided the labia minora photo into 10 equal sections and measured the width of each section. Yet, it is worth noting that taking genital photographs may not be practical or preferred by all participants due to individual preferences and cultural considerations.^5^ A study conducted in Thailand used calipers to measure genital dimensions, revealing significant variation in the appearance of Southeast Asian women’s genitalia.^17^

One aspect that contributes to people’s desire for labiaplasty is the presence of asymmetry in the labia minora.^18^ Dogan and Yassa reported an average width of 1.2 ± 4.1 cm for the right labia and 1.57 ± 4 cm for the left labia.^19^ In our study, the widest part of the labia minora on the right side had an average width of 1.67 cm, and on the left side, it was 1.76 cm.

Research findings have brought to light that a significant number of women who consider labiaplasty experience physical and appearance-related symptoms. These symptoms often manifest as discomfort during intercourse and while wearing tight pants, among others.^20^ In support of this, various studies have reported a high satisfaction rate and relatively low complication rate associated with labiaplasty.^14^^,^^21^ These positive outcomes have subsequently encouraged surgeons to explore and provide different methods for performing the procedure, aiming to cater to the diverse needs and preferences of patients.^17^^,^^22^

Additionally, Sasson et al argued that due to the sensitive and private nature of the body area involved in labiaplasty and the procedure’s similarity to female circumcision, external influences such as third-party opinions cannot easily sway women. This suggests that factors beyond external influences, such as personal emotions and considerations, play a significant role in a woman’s decision.^23^

Two earlier studies suggested that labiaplasty may be an unnecessary procedure, and the concern for atrophy of the labia minora during menopause should be warned to labiaplasty applicants. These studies argue that during menopause, the labia may experience atrophy as a result of decreased estrogen levels. To maintain a normal and healthy sexual response, it is posited that menopausal women require labial tissue that has an intact blood supply and innervation.^18^^,^^24^^,^^25^

Furthermore, other studies have highlighted the importance of educating women before undergoing FGCS, specifically about the normal variations in labia. By providing comprehensive information, women can gain a better understanding of their bodies and make informed decisions about potential surgical interventions. However, researchers continue to debate the normal size of the labia minora, external genital dimensions in women, and the practice of labiaplasty as a form of FGCS. The topic of labia hypertrophy and its impact on women’s sexual performance remains contentious within the scientific community.^3^^,^^15^^,^^16^^,^^26^^,^^27^

Additionally, the incorporation of psychological and sexual counseling as part of the preoperative process has been suggested. Gynecologists can also play a crucial role in effectively guiding women through this process, offering their expertise in addressing their concerns.^22^^,^^28^

In the present study, the dimensions of the external genitalia and 4 transverse distances on each side of the labia minora were measured by a gynecologist using a disposable paper measuring tape, which was a cost-effective and noninvasive method. This approach preserved participants’ privacy by not taking photographs and kept the study cost-effective, which are strengths of our research. Documenting the dimensions of the female external genitalia and long-term follow-up in case of surgery can help the applicant for FGCS make a better decision to choose and accept the complications of elective surgery.

Although the exact number of labiaplasty procedures performed in Iran is not available, the present study is the only one that shows the size of the external genitalia of women in Iran. One of the weaknesses of our study is the small number of samples. We are trying to conduct a larger study to measure the genital dimensions of Iranian women via a valid questionnaire, and this study will help us as the initial database and pilot Labiagram. Another limitation is not checking the amount of pelvic organ prolapse. This study was conducted to present the dimensions of the external genital organs of Iranian women who are not applicants for genital cosmetic surgery. However, if these parameters are presented among the people applying for FGCS, knowing the prolapse rate can better check the motivation of people applying for surgery.

## Conclusion

The use of Labiagram can be of great help in promoting the research goals of FGCS in countries that are culturally and religiously limited to providing pictures of the external genitalia of women.

The present study provides data on the average dimensions of the labia minora and external genitalia dimensions of Iranian women and the causes related to their size changes. This topography can help surgeons and women applying for genital cosmetic surgery know the extent of genital types. Also, this study will be the first step for other evaluations, such as indications, contraindications, and complications of FGCS, even the relationship between genital appearance and psychological and sexual factors in Iran.

## Author contributions

Z.G.H., M.K., and A.Z. conceived and designed the evaluation and drafted the manuscript. Z.G.H., M.K., S.S.H., and A.Z. designed and prepared the Labiagram chart on the web platform. Z.G.H., M.K., N.E., and A.Z. participated in designing the evaluation, performed parts of the statistical analysis, and helped to draft the manuscript. Z.G.H., M.K., N.E., S.S.H., and A.Z. reevaluated the clinical data, revised the manuscript and performed the statistical analysis, and revised the manuscript. Z.G.H., M.K., and A.Z. collected the clinical data, interpreted them, and revised the manuscript. Z.G.H., N.E., S.S.H., and A.Z. reanalyzed the clinical and statistical data and revised the manuscript. All authors read and approved the final manuscript.

## Funding

This research did not receive any specific grant from funding agencies in the public, commercial, or not-for-profit sector.

## Conflicts of interest

No financial aid was received from any institution, organization, or person to conduct this study.
